# Impacts of RETN genetic polymorphism on breast cancer development

**DOI:** 10.7150/jca.38088

**Published:** 2020-02-20

**Authors:** Chao-Qun Wang, Chih-Hsin Tang, Huey-En Tzeng, Lulu Jin, Jin Zhao, Le Kang, Yan Wang, Gui-Nv Hu, Bi-Fei Huang, Xiaoni Li, Yong-Ming Zhao, Chen-Ming Su, Hong-Chuan Jin

**Affiliations:** 1 Laboratory of Cancer Biology, Key Laboratory of Biotherapy in Zhejiang Province, Sir Run Run Shaw Hospital, Medical School of Zhejiang University, Hangzhou, China; 2 Department of Pathology, Affiliated Dongyang Hospital of Wenzhou Medical University, Dongyang, Zhejiang, China; 3 Department of Pharmacology, School of Medicine, China Medical University, Taichung, Taiwan; 4 Chinese Medicine Research Center, China Medical University, Taichung, Taiwan; 5 Department of Biotechnology, College of Health Science, Asia University, Taichung, Taiwan; 6 Taipei Cancer Center, Taipei Medical University, Taipei, Taiwan; 7 Graduate Institute of Cancer Biology and Drug Discovery, College of Medical Science and Technology, Taipei Medical University, Taipei, Taiwan; 8 Division of Hematology/Oncology, Department of Medicine, Taipei Medical University-Shuang Ho Hospital, Taiwan; 9 Department of Biomedical Sciences Laboratory, Affiliated Dongyang Hospital of Wenzhou Medical University, Dongyang, Zhejiang, China; 10 Department of Medical Oncology, Affiliated Dongyang Hospital of Wenzhou Medical University, Dongyang, Zhejiang, China; 11 Department of Surgical Oncology, Affiliated Dongyang Hospital of Wenzhou Medical University, Dongyang, Zhejiang, China; 12 Hefei National Laboratory for Physical Sciences at Microscale and School of Life Sciences, University of Science and Technology of China, Anhui, China; 13 Department of Sports Medicine, China Medical University, Taichung, Taiwan

**Keywords:** single nucleotide polymorphism, breast cancer, resistin, The Cancer Genome Atlas

## Abstract

The adipokine resistin is linked with obesity, inflammation and various cancers, including breast cancer. This study sought to determine whether certain polymorphisms in the gene encoding resistin, RETN, increase the risk of breast cancer susceptibility. We analyzed levels of resistin expression in breast cancer tissue and samples from The Cancer Genome Atlas database. We also examined associations between four RETN single nucleotide polymorphisms (SNPs; rs3745367, rs7408174, rs1862513 and rs3219175) and breast cancer susceptibility in 515 patients with breast cancer and 541 healthy women without cancer. Compared with wild-type (GG) carriers, those carrying the AG genotype of the RETN SNP rs3219175 and those carrying at least one A allele in the SNP rs3219175 had a higher chance of developing breast cancer (adjusted odds ratio, AOR: 1.295, 95% confidence intervals, CI: 1.065-1.575 and 2.202, 1.701-2.243, respectively). When clinical aspects and the RETN SNP rs7408174 were examined in the breast cancer cohort, the CT genotype was linked to late-stage disease, while women with luminal A disease and at least one C allele were likely to progress to stage III/IV disease and to develop highly pathological grade III disease. Moreover, resistin-positive individuals were at greater risk than resistin-negative individuals for developing pathological grade III disease (OR: 5.020; 95% CI: 1.380-18.259). This study details risk associations between resistin and RETN SNPs in breast cancer susceptibility in Chinese Han women.

## Introduction

GLOBOCAN 2018 estimates of cancer incidence and mortality document breast cancer as the most often diagnosed cancer that affects women, accounting for 11.6% of the total cancer cases worldwide 1. The risk of developing breast cancer is modified by various factors including age, reproductive and gynecological factors, physical activity, consumption of alcohol and tobacco, as well as family history 2 and gynecological diseases such as adenomyosis and polycystic ovarian syndrome 3, 4.

Genetic testing and mammography screening have limited specificity and sensitivity for evaluating an individual's level of breast cancer risk 2. Research indicates that single nucleotide polymorphism (SNP) genotyping might better predict an individual's risk for breast cancer and guide disease management 5, 6. Certain SNPs influence susceptibility to breast cancer 7. The risk of breast cancer is higher in those carrying the *BRCA1* and *BRCA2* gene mutations 8, 9 and genetic polymorphisms such as high-mobility group box protein 1 (HMGB1) and fascin-1 (FSCN1) 10, 11.

Resistin is a small cysteine-rich adipokine secreted by adipose tissue or constitutively secreted by macrophages 12; positive correlations have been observed between levels of plasma resistin and inflammatory markers, in addition to coronary artery calcification, a risk factor for coronary atherosclerosis 13. SNPs are found in the *RETN* promoter and 3'-untranslated regions 14. Genetic variation at the *RETN* locus carries a risk of several diseases, including the metabolic syndrome and colon cancer 15, 16 and those with a functional *RETN* gene polymorphism at -420 (rs1862513) are at risk of developing type 2 diabetes 17, 18, and associated with obesity in Tunisian population 19. *RETN* SNPs have been found to correlated with worsening disease in Chinese Han patients with lung cancer 20. There is *in vitro* evidence of upregulated *RETN* gene expression in samples of human breast cancer tissue 21 and polycystic ovary syndrome 22, but up until now, no association has been observed between *RETN* gene polymorphisms and breast cancer prognosis. Here we investigated some RETN SNPs with higher impact or risks in various cancers. This case-control study examined the involvement of four *RETN* SNPs and clinicopathological features in susceptibility to breast cancer amongst women of Chinese Han ethnicity.

## 2. Materials and Methods

### 2.1. Participants

This study enrolled 515 Chinese Han women with breast cancer (cases) presenting to Dongyang People's Hospital (Dongyang, Zhejiang, China) and 541 healthy, community-dwelling women without cancer (controls) between 2014 and 2018; all participants provided one blood sample with 3─5 mL. We also enrolled 154 untreated women scheduled for breast cancer surgery at the Affiliated Dongyang Hospital of Wenzhou Medical University (Dongyang, Zhejiang, China) between 2007 and 2017; one tissue specimen was obtained from each participant. Tumor grades were assigned using the Scarff-Bloom-Richardson system and the World Health Organization breast tumor classification criteria were used for pathohistological diagnosis 23 Cases were assigned estrogen receptor (ER), progesterone receptor (PR), human epidermal growth factor receptor 2 (HER2) and Ki-67 status and subtyped as Luminal A (ER-positive [+] and/or PR^+^, HER2-negative [-], Ki-67 <14%), Luminal B (ER^+^ and/or PR^+^, HER2^-^, Ki-67 ≥14%, ER^+^ and/or PR^+^, HER2^+^), HER2-enriched (ER^-^, PR^-^, HER2^+^), or as triple-negative breast cancer (TNBC; ER^-^, PR^-^, HER2^-^) 24,25,26. Clinicopathological information was collected from electronic medical records and at study entry each study participant completed a standardized questionnaire providing sociodemographic data. The study protocol was approved by the Dongyang People's Hospital Ethics Committee and all study procedures complied with guidelines and regulations as required. All study participants supplied fully informed written consent at the time of study entry.

### 2.2. Genotype determination

Following the manufacturer's instructions, we used QIAamp DNA blood mini kits (Qiagen, Valencia, CA) to isolate total genomic DNA from whole blood specimens. TE buffer (1 mM EDTA and 10 mM Tris pH 7.8) was used to dissolve DNA, which was stored at -20°C until quantitative polymerase chain reaction (qPCR) analysis. Four *RETN* SNPs were selected for analysis (rs3745367, rs7408174, rs1862513 and rs3219175), as they have previously been found to correlate with breast cancer progression 20 SNPs were genotyped by the TaqMan SNP genotyping assay (Applied Biosystems, Warrington, UK), according to the manufacturer's protocol 27,28. qPCRs were performed as previously described in a total volume of 20 μL containing a specific Master Mix (10 μL), probes (0.5 μL) and 10 ng of individual genomic DNA 24. Real-time PCR was performed as previously described, including an initial denaturation step at 95°C for 10 min, then 40 amplification cycles at 95°C for 15 secs and 60°C for 1 min 24,29.

### 2.3. Bioinformatics analysis

Data from an independent cohort of 1,904 breast cancer samples from The Cancer Genome Atlas (TCGA) database 30 were analyzed for overall and disease-free survival using Kaplan-Meier analysis and for gene expression data using the Bioconductor edgeR package (version 3.5.1) (https://www.r-project.org/). Patient profiles that were lacking relevant information were excluded before each analysis.

Correlations between SNPs and levels of *RETN* expression were identified using genotype-tissue expression (GTEx) data 24, 31. Expression of quantitative trait loci (eQTL) was analyzed to determine the functional role of phenotype-associated SNPs.

### 2.4. Immunohistochemistry

The Department of Pathology in Dongyang People's Hospital supplied human breast cancer tissue arrays including 154 breast cancer tissue specimens and 42 normal, cancer-free tissue specimens. Human breast cancer tissue was rehydrated and incubated with 3% hydrogen peroxide to quench endogenous peroxidase activity, then blocked by 3% BSA incubation in phosphate-buffered saline (PBS). After overnight incubation at 4°C with primary mouse anti-human resistin antibody (1:200 dilution), the tissue sections underwent 3 PBS washes and then staining with biotin-labeled secondary antibody and detection with the ABC kit (Vector Laboratories, Burlingame, CA, USA). Slides were stained with chromogen diaminobenzidine, washed, counterstained with Delafield's hematoxylin, dehydrated, treated with xylene then mounted. Two pathologists independently scored each slide for the amount of staining. Resistin expression and staining intensity were scored on a 4-point scale from 0 (no expression) to 1^+^ (weak), 2^+^ (moderate), or 3^+^ (strong).

### 2.5. Statistical analysis

Between-group differences were treated as significant when *p*-values were less than 0.05. The SNP genotype distributions were subjected to Chi-square testing for Hardy-Weinberg equilibrium. Demographic comparisons between cases and controls were analyzed using the Mann-Whitney U-test and Fisher's exact test. Since the data was independent and normal distribution, Fisher's exact test was used to compare differences in demographic characteristics between healthy controls and patients with breast cancer and Bonferroni's correction for multiple comparisons. Multiple logistic regression models accounting for confounding variables yielded adjusted odds ratios (AORs) and 95% confidence intervals (CIs) for associations between genotype frequencies and breast cancer or clinicopathological characteristics. All data were analyzed by the software program Statistical Product and Service Solutions (SPSS) version 19 and are indicated as the sample mean ± standard deviation (SD).

## 3. Results

**Figure 1:**
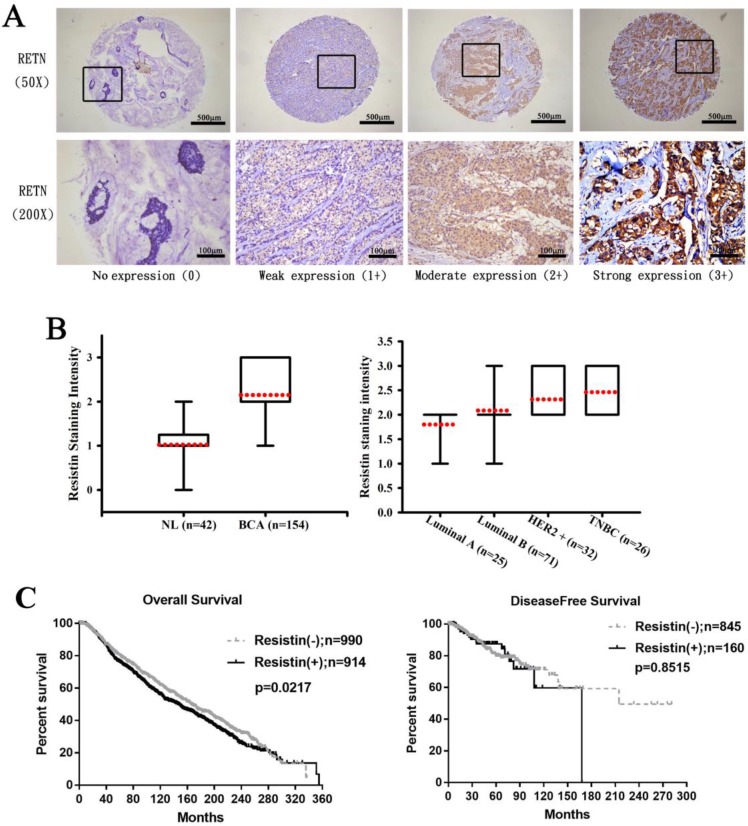
** Resistin expression levels in breast cancer patients. (A)** Breast cancer and normal tissue specimens were analyzed by IHC staining using resistin antibody. The stained specimens were photographed using an optical microscope and scored from 0-3 for levels of resistin expression. **(B)** Quantitative results of resistin expression in breast cancer specimens. **(C)** Kaplan-Meier analysis of overall and disease-free survival were compared with the resistin-negative and resistin-positive groups using the cancer genome atlas dataset. Patients profiles that were missing relevant information were excluded before each analysis.RETN, resistin; NL, normal tissues; BCA, breast cancer tissues; HER2, human epidermal growth factor receptor 2; TNBC, triple-negative breast cancer.

First, we analyzed clinical correlations between resistin expression and breast cancer. Immunohistochemistry (IHC) staining revealed higher resistin expression in the 154 tumor tissue specimens than in the 42 normal, healthy tissue specimens (Fig. 1A) and much stronger resistin staining in HER2^+^ and TNBC tumor subtypes than in luminal A and luminal B disease specimens (Fig. 1B), suggesting that resistin facilitates the progression of breast cancer. Kaplan-Meier analysis of the TCGA datasets revealed significantly poorer overall survival in the resistin-positive cohort (levels of resistin expression score of 3-4) compared to the resistin-negative group (score of 0-1); no such between-group difference was observed for disease-free survival (Fig. 1C).

All study participants were identified as Chinese Han ethnicity (Table 1). Most were nonsmokers (97.8%) and did not drink alcohol (95.9%). The mean age of the controls was significantly younger than that of the breast cancer cohort (40.47 years vs 53.11 years; p<0.05). Most patients (78%) had stage I/II breast cancer; 22% had stage III/IV disease (Table 1). Almost half (46.6%) had lymph node (N) N1-N3 metastasis. Nearly all tumors (96.9%) were classified as metastasis (M) M0 status (Table 1). Tumors were mostly ER^+^ (71.1%), PR^+^ (58.3%), or HER2^-^ (65.4%) (Table 1).

Table 2 depicts polymorphism frequencies. All genotypes were in Hardy-Weinberg equilibrium (p>0.05). In cases and controls, most of those with the rs3745367 SNP were homozygous for the GG genotype, most of those with the rs7408174 SNP were homozygous for the TT genotype, most of those with the rs1862513 SNP were homozygous for CC, and most of those with the rs3219175 SNP were homozygous for GG (Table 2).

In analyses that adjusted for confounders, study participants with the AG or the AG+AA genotype of the *RETN* rs3219175 polymorphism were around twice as likely to develop breast cancer as compared with those who were GG homozygous (AOR: 2.202; 95% CI: 1.701-2.243 and 1.869; 1.457-2.397, respectively; p<0.05 for both comparisons). In addition, those with the G allele of the *RETN* rs3219175 polymorphism were more likely than those with the A allele to develop breast cancer (AOR: 1.295; 95% CI: 1.065-1.575; p<0.05). Between-group differences were not significant for the proportions of breast cancer patients with the rs3745367, rs7408174 and rs1862513 polymorphisms, as compared with healthy controls (Table 2).

In a comparison of clinical aspects and rs7408174 *RETN* genotypes, patients with the CT genotype were almost twice as likely as those with the TT genotype to develop stage III/IV disease (OR: 1.725; 95% CI: 1.113-2.674), while those with at least one C allele were more likely to develop pathological grade III disease (Table 3).

When we analyzed the clinical aspects of rs7408174 and rs3219175 *RETN* genotypic frequencies among breast cancer subtypes, we found that among patients with the luminal A subtype, those carrying the CT genotype at SNP rs7408174 were much more likely than TT genotype carriers to develop stage III/IV disease and pathological grade II and III disease (OR: 3.084; 95% CI: 1.146-8.299 and 3.983; 1.531-10.362, respectively) (Table 4). In an analysis of resistin expression and clinical status in breast cancer tissue samples, high BMI (>24 kg/m^2^) and resistin positivity was associated with a 5-fold higher likelihood of pathological grade III disease as compared with resistin negativity (OR: 5.020; 95% CI: 1.380-18.259) (Table 5).

**Figure 2:**
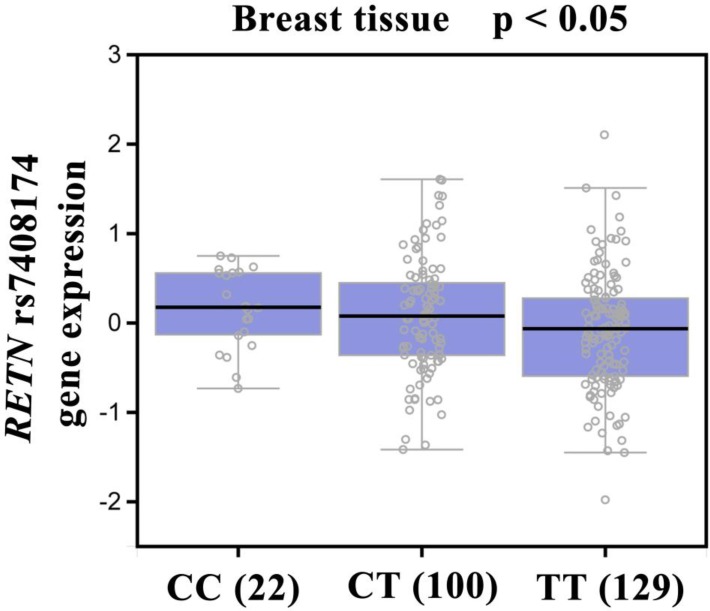
Correlation of rs7408174 genotype with *RETN* mRNA expression in breast cancer tissues in the Genotype-Tissue Expression (GTEx) dataset.

Our analysis of GTEx data revealed that individuals carrying the CC genotype of SNP rs7408174 showed a trend for increased resistin expression, compared with patients who had the wild-type TT homozygous genotype (p<0.05; Fig. 2).

## 4. Discussion

Not only is the adipokine resistin associated with obesity, inflammation, and various cancers 32,33,34, but high serum resistin levels have been implicated in the pathogenesis of cachexia in lung cancer 35, while resistin overexpression or upregulation is a feature of several human cancers, including oral cancer, renal cell carcinoma, chondrosarcoma and colon cancer 36,37,38,39. Notably, resistin helps to promote tumor growth, drug resistance and metastasis in breast cancer 40,41,42. A mechanical study has recently demonstrated the effect of resistin on epithelial to mesenchymal transition and stemness in breast cancer cells, which might be regulated by cyclase-associated protein 1 (CAP1) 43. Our IHC results confirmed higher levels of resistin expression in breast cancer specimens than in samples from cancer-free tissue and we found stronger resistin staining in tissue samples representing HER2^+^ and TNBC disease than in samples from luminal A and luminal B disease, while our analysis of TCGA data revealed significantly poorer overall survival in resistin-positive tissue compared to resistin-negative samples. Notably, inhibition of resistin reduces chondrosarcoma metastasis and lymphangiogenesis 32,36. Thus, the data suggest that therapeutic strategies that effectively inhibit resistin could be useful in breast cancer.

The prognosis of breast cancer patients depends on the clinical or pathological stage at diagnosis. Thus, individuals with hereditary breast cancer could benefit from epigenetic screening for early diagnosis and treatment that prevents the disease from developing. *RETN* polymorphisms have been identified in various cancers, including colon and lung 16,20,21, but data are scant as to the involvement of *RETN* polymorphisms in breast cancer. As far as we are aware, our study is the first to investigate the distributions of the rs3745367, rs7408174, rs1862513 and rs3219175 SNPs and their associations with the development and progression of breast cancer in Chinese Han women. Here, we found that women carrying the GG genotype of the *RETN* rs3219175 polymorphism were more likely than those with GG homozygotes to develop breast cancer, while those carrying at least one A allele in rs3219175 compared with carriers of wild-type GG homozygotes were at increased risk for breast cancer. Our previous study has reported SNP rs3219175 and rs7408174 at greater risk of developing RA disease 44, and the effects of these variants on resistin expression require to be further examined in breast cancer cells in the future. A previous study has reported that the rs1862513 SNP in *RETN* increased breast cancer risk and a tendency for luminal breast tumors in Mexican women, which divided into different subtypes according to BMI 45. It was similar to our results but we separated different subtypes of breast cancer patients based on their clinical pathohistological diagnosis, and we could further considered BMI variable and metastatic gene *CAP1* in our future study. This evidence implicates critical roles for resistin and *RETN* polymorphisms in breast cancer.

Between 2010 and 2014, 5-year relative survival rates for breast cancer were ~90.2% in the USA 46 and ~80% in China 47. As the prognosis of breast cancer patients depends on their clinical and pathological status at diagnosis, early diagnosis is essential and is becoming ever more possible with improvements in screening strategies and the wider availability of epigenetic strategies 48, 49. We investigated possible associations between *RETN* polymorphisms, clinical and pathological markers, and susceptibility to breast cancer. We found that individuals carrying the CT genotype at the rs7408174 polymorphism were more likely to progress to late-stage disease. Carriers of at least one C allele at rs7408174 had a higher risk of developing stage III/IV disease and of developing highly pathological grade III disease. Similarly, among those with luminal A breast cancer, having the CT genotype at SNP rs7408174 was linked to a risk of stage III/IV disease and pathological grade III disease. The SNP rs3219175 had no significance in the clinical status of breast cancer patients (data not shown). Our results also revealed that high BMI (>24 kg/m^2^) in the resistin-positive cohort increased the risk of pathological grade III disease. Our findings contribute to data concerning the correlation between resistin and breast cancer development. In addition, no significant patterns of linkage disequilibrium were observed in any of the *RETN* genotypes analysed from breast cancer patients (Supplementary Fig. S1).

It is established that gene expression can be controlled by polymorphisms that appear in the 3ʹ-flanking region 50. According to the evidence from the GTEx database, the CC genotype at rs7408174 showed a trend for increased expression of resistin, compared with levels of resistin expression found in individuals with wild-type TT homozygous genotypes. This result confirms our SNP data and indicates that *RETN* SNPs rs7408174 and rs3219175 SNPs may control resistin expression.

In conclusion, our investigation demonstrates an association between *RETN* gene variants and susceptibility for breast cancer and its progression among Chinese Han women carrying the *RETN* rs3219175 and rs7408174 polymorphisms. We also identified high levels of resistin expression in breast cancer patients. Resistin appears to be a predictive marker for breast cancer treatment.

## Supplementary Material

Supplementary figure S1.

## Figures and Tables

**Table 1:** Demographic and clinicopathological characteristics of healthy controls and patients with breast cancer.

Variable	Controls n=541 (%)	Patients n=515 (%)	*p* value
**Age (years)**	Mean ± S.D.	Mean ± S.D.	
	40.47 ± 15.61	53.11 ± 11.64	*p* < 0.05
**Cigarette smoking**			
No	529 (97.8)	514 (99.8)	
Yes	12 (2.2)	1 (0.2)	*p* < 0.05
**Alcohol consumption**			
No	519 (95.9)	481 (93.4)	
Yes	22 (4.1)	34 (6.6)	*p* > 0.05
**Clinical stage**			
I+II		403 (78.3)	
III+IV		112 (21.7)	
**Tumor size**			
≤T2		482 (93.6)	
>T2		33 (6.4)	
**Lymph node status**			
N0		275 (53.4)	
N1+N2+N3		240 (46.6)	
**Distant metastasis**			
M0		499 (96.9)	
M1		16 (3.1)	
**Pathological grade**			
I		51 (9.9)	
II		306 (59.4)	
III		158 (30.7)	
**ER status**			
Negative		149 (28.9)	
Positive		366 (71.1)	
**PR status**			
Negative		215 (41.7)	
Positive		300 (58.3)	
**HER2 status**			
Negative		337 (65.4)	
Positive		178 (34.6)	
**Ki-67** ≤14%		152 (29.5)	
>14%		363 (70.5)	

The Mann-Whitney U test or Fisher's exact test was used to compare values between controls and patients with breast cancer. ER, estrogen receptor; PR, progesterone receptor; HER2, human epidermal growth factor receptor 2. Pathological grade: I, well differentiated; II, moderately differentiated; III, poorly differentiated.

**Table 2:** Distribution frequencies of *RETN* genotypes in controls and patients with breast cancer.

Variable	Controls n=541 (%)	Patients n=515 (%)	OR (95% CI)	AOR (95% CI)
rs3745367				
GG	212 (39.2)	184 (35.7)	1.00 (reference)	1.00 (reference)
AG	252 (46.6)	259 (50.3)	1.184 (0.910-1.540)	1.180 (0.904-1.542)
AA	77 (14.2)	72 (14.0)	1.077 (0.739-1.571)	1.100 (0.751-1.609)
AG+AA	329 (60.8)	331 (64.3)	1.159 (0.903-1.488)	1.163 (0.903-1.499)
G	676 (62.5)	627 (60.9)	1.00 (reference)	1.00 (reference)
A	406 (37.5)	403 (39.1)	1.070 (0.898-1.276)	1.080 (0.904-1.291)
rs7408174				
TT	311 (57.5)	295 (57.3)	1.00 (reference)	1.00 (reference)
CT	196 (36.2)	186 (36.1)	1.000 (0.774-1.293)	1.005 (0.775-1.303)
CC	34 (6.3)	34 (6.6)	1.054 (0.639-1.740)	1.040 (0.625-1.728)
CT+CC	230 (42.5)	220 (42.7)	1.008 (0.790-1.287)	1.010 (0.788-1.294)
T	818 (75.6)	776 (75.3)	1.00 (reference)	1.00 (reference)
C	264 (24.4)	254 (24.7)	1.014 (0.832-1.237)	1.012 (0.828-1.238)
rs1862513				
CC	224 (41.4)	214 (41.6)	1.00 (reference)	1.00 (reference)
CG	241 (44.5)	205 (39.8)	0.890 (0.684-1.160)	0.887 (0.677-1.160)
GG	76 (14.0)	96 (18.6)	1.322 (0.928-1.885)	1.363 (0.952-1.950)
CG+GG	317 (58.6)	301 (58.4)	0.994 (0.778-1.270)	1.003 (0.782-1.286)
C	689 (63.7)	633 (61.5)	1.00 (reference)	1.00 (reference)
G	393 (36.3)	397 (38.5)	1.110 (0.922-1.312)	1.118 (0.935-1.338)
rs3219175				
GG	316 (58.4)	226 (43.9)	1.00 (reference)	1.00 (reference)
AG	183 (33.8)	277 (53.8)	2.116 (1.643-2.726)*	2.202 (1.701-2.851)*
AA	42 (7.8)	12 (2.3)	0.399 (0.206-0.776)*	0.418 (0.214-0.817)*
AG+AA	225 (41.6)	289 (56.1)	1.796 (1.407-2.292)*	1.869 (1.457-2.397)*
G	815 (75.3)	729 (70.8)	1.00 (reference)	1.00 (reference)
A	267 (24.7)	301 (29.2)	1.260 (1.039-1.528)*	1.295 (1.065 -1.575)*

The odds ratios (ORs) and their associated 95% confidence intervals (CIs) were estimated by logistic regression analysis. The adjusted odds ratios (AORs) with their associated 95% CIs were estimated by multiple logistic regression analysis that controlled for alcohol consumption and age >55 years. * *p* < 0.05 was considered to be statistically significant.

**Table 3:** Odds ratios (ORs) and 95% confidence intervals (CIs) of clinical status and *RETN* rs7408174 genotypic frequency in 515 patients with breast cancer.

Gene Genotypes	Patients		OR (95% CI)	AOR (95% CI)
**Clinical Stage**				
	Stage I/II	Stage III/IV		
rs7408174	n=403 (%)	n=112 (%)		
TT	242 (60.0)	53 (47.3)	1.00 (reference)	1.00 (reference)
CT	135 (35.5)	51 (45.5)	1.725 (1.113-2.674)*	1.715 (1.106-2.659)*
CC	26 (6.5)	8 (7.1)	1.405 (0.603-3.275)	1.414 (0.605-3.309)
CT+CC	161 (40.0)	59 (52.7)	1.673 (1.098-2.549)*	1.664 (1.092-2.537)*
**Tumor size**				
	≤T2	>T2		
rs7408174	n=482 (%)	n=33 (%)		
TT	278 (57.9)	16 (48.5)	1.00 (reference)	1.00 (reference)
CT	173 (35.9)	13 (39.4)	1.310 (0.615-2.791)	1.296 (0.608-2.764)
CC	30 (6.2)	4 (12.1)	2.325 (0.730-7.406)	2.279 (0.713-7.284)
CT+CC	203 (42.1)	17 (51.5)	1.460 (0.721-2.959)	1.451 (0.716-2.943)
**Lymph node metastasis**				
	N0	N1+N2+N3		
rs7408174	n=275 (%)	n=240 (%)		
TT	163 (59.3)	85 (55.0)	1.00 (reference)	1.00 (reference)
CT	90 (32.7)	96 (40.0)	1.317 (0.912-1.903)	1.314 (0.908-1.900)
CC	22 (8.0)	12 (5.0)	0.674 (0.321-1.412)	0.672 (0.320-1.411)
CT+CC	112 (40.7)	108 (45.0)	1.191 (0.839-1.690)	1.184 (0.833-1.681)
**Distant metastasis**				
	M0	M1		
rs7408174	n=499 (%)	n=16 (%)		
TT	287 (57.5)	8 (50.0)	1.00 (reference)	1.00 (reference)
CT	181 (36.3)	5 (31.3)	0.991 (0.319-3.076)	0.986 (0.315-3.086)
CC	31 (6.2)	3 (18.7)	3.472 (0.875-13.768)	3.241 (0.807-13.026)
CT+CC	212 (42.5)	8 (50.0)	1.354 (0.500-3.665)	1.332 (0.488-3.637)
**Pathological grade**				
	I+II	III		
rs7408174	n=357 (%)	n=158 (%)		
TT	215 (60.2)	80 (50.6)	1.00 (reference)	1.00 (reference)
CT	125 (35.0)	61 (38.6)	1.312 (0.880-1.956)	1.322 (0.886-1.972)
CC	17 (4.8)	17 (10.8)	2.688 (1.309-5.519)*	2.759 (1.339-5.685)*
CT+CC	142 (39.8)	78 (49.4)	1.476 (1.012-2.152)*	1.484 (1.017-2.164)*

The ORs with their 95% CIs were estimated by logistic regression models. The adjusted odds ratios (AORs) with their 95% CIs were estimated by multiple logistic regression models that controlled for alcohol consumption and age. * *p* < 0.05 was considered to be statistically significant. Pathological grade: I, well differentiated; II, moderately differentiated; III, poorly differentiated.

**Table 4:** Odds ratios (ORs) and 95% confidence interval (CIs) of *RETN* genotypic frequencies and clinical subtypes in patients with breast cancer.

Variable	Luminal A (n=143)			Luminal B (n=224)			HER2 overexpression (n=80)			TNBC (n=68)		
	Clinical stage		OR (95% CI)	Clinical stage		OR (95% CI)	Clinical stage		OR (95% CI)	Clinical stage		OR (95% CI)
	Stage I/II	Stage III/IV		Stage I/II	Stage III/IV		Stage I/II	Stage III/IV		Stage I/II	Stage III/IV	
rs7408174	N=123(%)	N=20(%)		N=169(%)	N=55(%)		N=55(%)	N=25(%)		N=56(%)	N=12(%)	
TT	83 (67.5)	8 (40.0)	1.00 (reference)	102 (60.4)	28 (50.9)	1.00 (reference)	31 (56.4)	12 (48.0)	1.00 (reference)	26 (46.4)	5 (41.7)	1.00 (reference)
CT	37 (30.1)	11 (55.0)	3.084 (1.146-8.299)*	57 (33.7)	23 (41.8)	1.470 (0.775-2.787)	17 (30.9)	10 (40.0)	1.520 (0.544-4.243)	24 (34.4)	7 (58.3)	1.517 (0.424-5.426)
CC	3 (2.4)	1 (5.0)	3.458 (0.321-37.242)	10 (5.9)	4 (7.3)	1.457 (0.425-4.998)	7 (12.7)	3 (12.0)	1.107 (0.245-5.000)	6 (10.7)	0 (0)	—
CT+CC	40 (32.5)	12 (60.0)	3.113 (1.179-8.218)*	67 (39.6)	16 (49.1)	1.468 (0.796-2.707)	24 (43.6)	13 (52.0)	1.399 (0.542-3.613)	30 (53.6)	7 (58.3)	1.213 (0.343-4.286)
	Tumor size		OR (95% CI)	Tumor size		OR (95% CI)	Tumor size		OR (95% CI)	Tumor size		OR (95% CI)
	≦T2	> T2		≦T2	> T2		≦T2	> T2		≦T2	> T2	
rs7408174	N=140(%)	N=3(%)		N=208(%)	N=16(%)		N=71(%)	N=9(%)		N=63(%)	N=5(%)	
TT	90 (64.3)	1 (33.3)	1.00 (reference)	122 (58.7)	8 (50.0)	1.00 (reference)	38 (53.5)	5 (55.6)	1.00 (reference)	29 (46.0)	2 (40.0)	1.00 (reference)
CT	46 (32.9)	2 (66.7)	3.913 (0.346-44.297)	74 (35.6)	6 (37.5)	1.236 (0.413-3.704)	25 (35.2)	2 (22.2)	0.608 (0.109-3.381)	28 (44.4)	3 (60.0)	1.554 (0.241-10.010)
CC	4 (2.9)	0 (0)	—	12 (5.7)	2 (12.5)	2.542 (0.484-13.355)	8 (11.3)	2 (22.2)	1.900 (0.311-11.591)	6 (9.6)	0 (0)	—
CG+GG	50 (35.7)	2 (66.7)	3.600 (0.318-40.697)	86 (41.3)	8 (50.0)	1.419 (0.513-3.927)	30 (58.8)	4 (44.4)	0.921 (0.228-3.717)	34 (54.0)	3 (60.0)	1.279 (0.200-8.190)
	Lymph node status		OR (95% CI)	Lymph node status		OR (95% CI)	Lymph node status		OR (95% CI)	Lymph node status		OR (95% CI)
	N0	N1-N3		N0	N1-N3		N0	N1-N3		N0	N1-N3	
rs7408174	N=91(%)	N=52(%)		N=111(%)	N=113(%)		N=35(%)	N=45(%)		N=38(%)	N=30(%)	
TT	61 (67.0)	30 (57.7)	1.00 (reference)	67 (60.4)	63 (55.8)	1.00 (reference)	20 (57.1)	23 (51.1)	1.00 (reference)	15 (39.5)	16 (53.3)	1.00 (reference)
CT	27 (29.7)	21 (40.4)	1.581 (0.771-3.244)	35 (31.5)	45 (39.8)	1.367 (0.781-2.393)	11 (31.4)	16 (35.6)	1.265 (0.478-3.349)	17 (44.7)	14 (46.7)	0.772 (0.285-2.095)
CC	3 (3.3)	1 (1.9)	0.678 (0.068-6.794)	9 (8.1)	5 (4.4)	0.591 (0.188-1.859)	4 (11.5)	6 (13.3)	1.304 (0.322-5.289)	6 (15.8)	0 (0)	—
CG+GG	30 (33.0)	22 (42.3)	1.491 (0.739-3.011)	44 (39.6)	49 (44.2)	1.209 (0.710-2.056)	15 (42.9)	22 (48.9)	1.275 (0.524-3.102)	23 (60.5)	14 (46.7)	0.571 (0.217-1.503)
	Distant metastasis		OR (95% CI)	Distant metastasis		OR (95% CI)	Distant metastasis		OR (95% CI)	Distant metastasis		OR (95% CI)
	M0	M1		M0	M1		M0	M1		M0	M1	
rs7408174	N=141(%)	N=2(%)		N=219(%)	N=5(%)		N=75(%)	N=5(%)		N=64(%)	N=4(%)	
TT	90 (63.8)	1 (50.0)	1.00 (reference)	127 (58.0)	3 (60.0)	1.00 (reference)	42 (56.0)	1 (20.0)	1.00 (reference)	28 (43.8)	3 (75.0)	1.00 (reference)
CT	47 (33.3)	1 (50.0)	1.915 (0.117-31.306)	79 (36.1)	1 (20.0)	0.536 (0.055-5.242)	25 (33.3)	2 (40.0)	3.360 (0.290-38.975)	30 (46.9)	1 (25.0)	0.311 (0.031-3.169)
CC	4 (2.8)	0 (0)	—	13 (5.9)	1 (20.0)	3.256 (0.316-33.604)	8 (10.7)	2 (40.0)	10.500 (0.848-130.072)	6 (9.3)	0 (0)	—
CG+GG	51 (36.2)	1 (50.0)	1.765 (0.108-28.818)	92 (42.0)	2 (40.0)	0.920 (0.151-5.619)	33 (44.0)	4 (80.0)	5.091 (0.543-47.736)	36 (56.2)	1 (25.0)	0.259 (0.026-2.629)
	Pathologic grade		OR (95% CI)	Pathologic grade		OR (95% CI)	Pathologic grade		OR (95% CI)	Pathologic grade		OR (95% CI)
	I	II+III		I	II+III		I	II+III		I	II+III	
rs7408174	N=41(%)	N=102(%)		N=7(%)	N=217(%)		N=2(%)	N=78(%)		N=1(%)	N=67(%)	
TT	33 (80.5)	58 (56.9)	1.00 (reference)	5 (71.4)	125 (57.6)	1.00 (reference)	1 (50.0)	42 (53.8)	1.00 (reference)	0 (0)	31 (46.3)	1.00 (reference)
CT	6 (14.6)	42 (41.1)	3.983 (1.531-10.362)*	22 (28.6)	78 (35.9)	1.560 (0.295-8.238)	1 (50.0)	26 (33.3)	0.619 (0.037-10.330)	1 (100.0)	30 (44.7)	—
CC	2 (4.9)	2 (2.0)	0.569 (0.077-4.229)	0 (0)	14 (6.5)	—	0 (0)	10 (12.8)	—	0 (0)	6 (9.0)	—
CT+CC	8 (19.5)	44 (43.1)	3.129 (1.316-7.440)*	22 (28.6)	92 (42.4)	1.840 (0.349-9.694)	1 (50.0)	36 (46.2)	0.857 (0.052-14.199)	1 (100.0)	36 (53.7)	—

The ORs and their associated 95% CIs were estimated by logistic regression models. * *p* < 0.05 was considered to be statistically significant. HER2, human epidermal growth factor receptor 2; TNBC, triple-negative breast cancer. Pathological grade: I, well differentiated; II, moderately differentiated; III, poorly differentiated.

**Table 5:** Comparison of the clinical parameters and resistin expression in 154 breast cancer tissue samples.

Parameter	Resistin-negative^a^ n=17 (%)	Resistin-positive^b^ n=137 (%)	OR (95% CI)
BMI			
	23.07 ± 2.42	24.19 ± 3.07	
Clinical stage			
I+II	9 (52.9)	103 (75.2)	
III+IV	8 (47.1)	34 (24.8)	0.371 (0.133-1.038)
Pathological grade			
I+II	14 (82.4)	66 (48.2)	
III	3 (17.6)	71 (51.8)	5.020 (1.380-18.259)*

^a^ Resistin-negative status was scored as 0 or 1; ^b^ Resistin-positive status was scored as 2 or 3. The odds ratios (ORs) and their associated 95% confidence intervals (CIs) were estimated by logistic regression models. *p < 0.05. BMI, body mass index.
