# Supplementary material for: Impacts of RETN genetic polymorphism on breast cancer development
Source: J Cancer. 2020 Feb 20;11(10):2769–77. doi: 10.7150/jca.38088 (PMC7086250; doi:10.7150/jca.38088)
Supplement: Supplementary file 1 — Supplementary figure S1. [file jcav11p2769s1.pdf]

Supplementary Figure legends

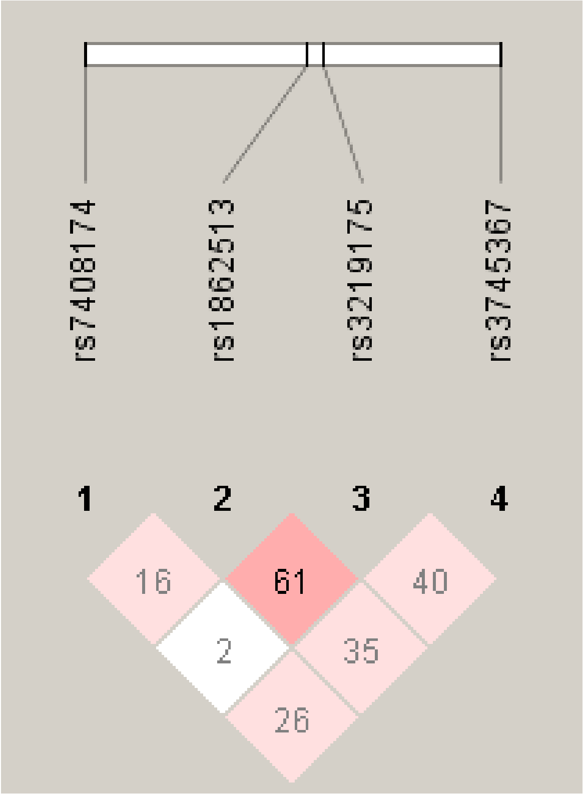

Figure S1. Linkage disequilibrium patterns of four single nucleotide polymorphisms in the *RETN* gene in Chinese han population.
